# Additive Effects of Item-Specific and Congruency Sequence Effects in the Vocal Stroop Task

**DOI:** 10.3389/fpsyg.2019.00860

**Published:** 2019-04-24

**Authors:** Andrew J. Aschenbrenner, David A. Balota

**Affiliations:** ^1^Department of Neurology, Washington University in St. Louis, St. Louis, MO, United States; ^2^Department of Psychological and Brain Sciences, Washington University in St. Louis, St. Louis, MO, United States

**Keywords:** attentional control, congruency sequence effect, item-specific proportion congruency effect, attention, cognitive control

## Abstract

There is a growing interest in assessing how cognitive processes fluidly adjust across trials within a task. Dynamic adjustments of control are typically measured using the congruency sequence effect (CSE), which refers to the reduction in interference following an incongruent trial, relative to a congruent trial. However, it is unclear if this effect stems from a general control mechanism or a distinct process tied to cross-trial reengagement of the task set. We examine the relationship of the CSE with another measure of control referred to as the item-specific proportion congruency effect (ISPC), the finding that frequently occurring congruent items exhibit greater interference than items that are often incongruent. If the two effects reflect the same control mechanism, one should find interactive effects of CSE and ISPC. We report results from three experiments utilizing a vocal Stroop task that manipulated these two effects while controlling for variables that are often confounded in the literature. Across three experiments, we observed large CSE and ISPC effects. Importantly, these effects were robustly additive with one another (Bayes Factor for the null approaching 9). This finding indicates that the CSE and ISPC arise from independent mechanisms and suggests the CSE in Stroop may reflect a more general response adjustment process that is not directly tied to trial-by-trial changes in attentional control.

## Introduction

Attentional control is the ability to select relevant attributes from the environment for additional processing while ignoring competing and possibly more salient attributes. The Stroop color naming task (Stroop, 1935) is a classic test of attentional selection. In this paradigm, individuals are presented with color words printed in colored ink (e.g., the word RED in blue ink) and are instructed to name the ink color and ignore the word. The degree to which responses to incongruent stimuli (where the color and word are different) are slower than responses to congruent stimuli (where the color and word are the same) reflects the efficiency of attentional control.

A key theoretical issue is how control is recruited and/or adjusted across trials within a task. Extant models have been informed by the robust finding that interference on Trial N is consistently smaller when the stimulus on Trial N-1 was incongruent relative to when that item was congruent (Gratton et al., 1992). This phenomenon is known as the congruency sequence effect (CSE). Importantly, the CSE indicates that some aspect of the stimulus from the prior trial induces a change in the processing system that influences performance on the subsequent trial(s). This suggests that attentional control is not a static process but rather is fluid and dynamic. A large body of research has since aimed to identify the specific mechanisms that produce these trial by trial adjustments in attentional control (see Duthoo et al., 2014b, for a review).

Many accounts of the CSE have been proposed and one of the most prominent is the conflict monitoring hypothesis which suggests the conflict produced by the stimulus on the preceding trial signals the system to upregulate control for the following trial (Botvinick et al., 2001). This theory has been able to account for a wide array of behavioral and neural data (Botvinick et al., 2004). Importantly, the conflict monitoring account suggests the CSE is fundamentally a modulation of control processes and has inspired a flurry of research that has aimed to determine whether the CSE truly reflects an adjustment in control. Some of the earliest alternative explanations suggested the CSE is actually produced by low-level feature characteristics such as item repetition (Mayr et al., 2003; Hommel et al., 2004) or response contingency (Schmidt and De Houwer, 2011). Although such confounds certainly do contribute to the observed effects, careful experimentation that has controlled for these confounds has generally still produced the expected finding, albeit reduced (Duthoo et al., 2014a; Kim and Cho, 2014; Schmidt and Weissman, 2014). Together these findings suggest that abstract properties (possibly conflict) of the prior stimulus are at least partially responsible for cross-trial changes and hence the CSE can be used as a marker of attentional control adjustment.

However, a number of studies have continued to challenge whether the CSE is a control phenomenon or rather arises from a more general trial-by-trial response adjustment mechanism. For example, Schmidt and Weissman (2016) conducted detailed analyses of prior trial response times and determined that the CSE is consistent with a simple temporal learning model. That is, participants tend to respond quickly after a relatively fast response (which tend to be congruent trials) on Trial N-1 and relatively slowly after a slow response (which tend to be incongruent trials) on Trial N-1. These expectations are implemented via momentary drops in response thresholds such that following a fast (congruent) trial, response thresholds are dropped relatively early and following a slow (incongruent) trial, thresholds are dropped relatively late. An early drop in threshold would benefit a congruent stimulus on Trial N whereas a later drop would benefit incongruent stimuli on Trial N, producing the CSE pattern (see Schmidt and Weissman, 2016, for computational details). It is important to point out, however, that while the statistical models revealed a robust current trial congruency by previous trial congruency by previous trial RT interaction (which indicates the CSE is modulated by the prior trial RT), the two-way interaction between current and previous congruency still remained. Thus, we can conclude that temporal learning may contribute to the magnitude of the CSE, but it is not the entire story.

Aschenbrenner and Balota (2015) took an individual differences approach and compared the magnitude of the CSE as a function of age and working memory in the Stroop task. They argued that because older adults and low-working memory individuals have impaired attentional control, one should expect these individuals to produce smaller CSEs. Instead, they found the opposite pattern, namely that the CSE *increased* with older age and lower working memory estimates. Furthermore, this increase was driven primarily by differences on post-congruent rather than post-incongruent trials.

The disproportionate influence of prior congruent responses (Lamers and Roelofs, 2011) led Aschenbrenner and Balota (2015) to propose a pathway priming account of the CSE. Specifically, they assumed a two-pathway model of Stroop performance (e.g., color and word pathway) in which activity accumulates along each pathway until a response is made. When Trial N-1 is incongruent, trials on which only the color dimension is relevant, the color pathway is primed for use on the subsequent trial. If Trial N is also incongruent, responses will be facilitated due to the greater activity along the color pathway. However, when Trial N-1 is congruent, the word pathway holds relative utility in reaching the correct response, hence primes the word pathway for use on the next trial. If Trial N is congruent, responses will again be facilitated due to increased activity along the word pathway, however if Trial N is incongruent, responses are slowed as the additional activity along the word pathway now needs to be controlled. Hence, the pathway priming model embodies the assumption that individuals are constantly adjusting specific procedures they utilize to achieve task goals based on the success of those procedures (e.g., use of color vs. word pathway) on the immediately preceding trial.

Of course, if this model is correct, then one should find cross trial effects in other tasks such as lexical decision and recognition memory, which are not tasks that place a heavy load on attentional control systems, certainly not to the same degree as the Stroop task. Indeed, there has been a recent flurry of research which suggests that non-attentional tasks also produce CSE-like patterns that can be interpreted within the pathway priming framework (Malmberg and Annis, 2012; Balota et al., 2018; Aschenbrenner et al., 2017; Hubbard et al., 2017).

As noted, most recent research has tried to address whether the CSE reflects control by eliminating all possible confounds (e.g., feature level characteristics) to ensure that some CSE is still obtained. We take an alternative approach here. Specifically, we examine these issues through the lens of the additive factors framework (Sternberg, 1969) which suggests that additive effects of two variables (i.e., reliable main effects but no interaction) indicate each variable influences a separate or independent processing stage whereas variables that interact influence a shared stage. For example, in the classic short-term memory scanning study where participants are shown a series of digits and asked to determine if a target probe is or is not contained in the presented array, it has been shown that the perceptual quality of the probe is additive with regards to the size of the memory set to be searched (Sternberg, 1967). Sternberg concluded that stimulus degradation and memory set size must each influence a separate processing stage. Of course, such an account is not the only way to interpret additive effects (e.g., McClelland, 1979), however the independent stages model has been shown to best accommodate the relationship among mean reaction times and the associated variances (Roberts and Sternberg, 1993, see Balota et al., 2013 for similar interpretation of the additivity of degradation and word frequency in the lexical decision task).

In the present study, we used additive factors logic to examine whether the CSE involves attentional control adjustments by exploring the relationship between the CSE and an established marker of attentional control adjustment, the item-specific proportion congruency effect (ISPC: Jacoby et al., 2003). Specifically, it has been repeatedly shown that the magnitude of interference on any given trial depends on the overall frequency with which that particular item is congruent or incongruent. That is, items which are mostly congruent (MC items) exhibit greater interference than items that are mostly incongruent (MI items). This finding has been interpreted as evidence for a rapid retrieval or adjustment of control settings that occurs post-stimulus onset (Blais et al., 2007). For example, if the word GREEN is typically incongruent, control over the word pathway would be increased when GREEN is encountered in the list. Using additive factors logic, if the CSE is due to an adjustment in control processes, then it should interact with the ISPC. In contrast, if the CSE is the result of some other, non-control based mechanism (such as pathway priming), one would expect additivity to prevail.

We conducted a modified vocal Stroop task in which the CSE was examined following biased ISPC items (i.e., mostly congruent or mostly incongruent) or unbiased (50% congruent) items. As already indicated, exact repetition of stimuli can artificially magnify the CSE and hence repetition of stimuli or responses should be precluded from the design. This is typically done by expanding the size of the stimulus set (e.g., by using at least four colors in the Stroop task). However, this standard manipulation produces another confound, specifically a contingency bias such that the word dimension predicts the correct response more often than would be expected by chance alone which can also influence the observed CSE (Schmidt and De Houwer, 2011).

Therefore, in order to provide a confound-minimized test of CSE processes in the current study, the following procedure was implemented (Kim and Cho, 2014; Aschenbrenner and Balota, 2017). First, we created a set of Stroop stimuli that consisted of eight colors and eight color words which were placed into pairs. Incongruent items were always shown in the color of the opposite item of the pair. For example, if RED and BLUE form one pair, an incongruent BLUE stimulus would always be shown in the color RED and never in any other color. Such a procedure eliminates the contingency confound, and as long as different pairs are sampled across adjacent trials exact repetitions of items and responses are also precluded.

As an overview of the experiments, Experiment 1 examined the relationship between the CSE and the ISPC in young adults using a vocal Stroop paradigm that eliminates all confounds that have been previously identified in the literature. Experiment 2 examined the same effects in a sample of older adult participants, a group of people who have been shown to have difficulties in attentional control and therefore should produce larger overall effects and may increase our power to detect interactive influences. Finally, Experiment 3 eliminated a potential alternative account of the ISPC (associative learning) to ensure that the present ISPC is indeed a reflection of attentional control in this paradigm.

## Experiment 1

### Methods

#### Participants

Thirty-two young adults (78% female; mean age = 19.7 years, *SD* = 1.4) were recruited from the Washington University Psychology undergraduate research pool. All had normal or corrected-to-normal vision and participated for research credit or monetary compensation. A power analysis using the Bayes factor design analysis (BFDA) package (Schönbrodt, 2018) in R indicated that a sample size of 32 would give approximately 70% power to obtain an interpretable Bayes factor (i.e., greater than three) in favor of a difference in the CSE as a function of the ISPC using a paired *t*-test, assuming a moderate effect size (Cohen’s D ranging from 0.45 to 0.65). Similarly, we had approximately 72% power to obtain a Bayes factor larger than three in favor of the null, assuming a true effect size of 0.

#### Stimuli

The stimulus set and the frequency of presentation of each item is shown in Table 1. The four pairs of items were presented with differing frequencies such that items from one pair were congruent 75% of the time (thus forming a mostly congruent: MC item set) and items from a different pair were only congruent 25% of the time (mostly incongruent: MI items). The final two pairs were 50% congruent, one of which was designated “neutral” items and the other as the “critical” items. The neutral items were intended to serve as a control condition to assess the CSE when the prior trial did not contain a frequency manipulation (consistent with prior examinations of the CSE). The critical items were used to assess the magnitude of the CSE. Importantly, while both the neutral and critical items are 50% congruent, only the critical items were experimentally controlled such that they followed each item type (MI, MC, and neutral) with equal probability. This insures that an equal number of trials occurred in each of the four cells that make up the CSE. The item pairs (e.g., RED always with BLUE) were kept the same but were rotated through the conditions such that each set of items was a MI, MC, neutral, or critical item across participants.

**Table 1:** Stimuli frequencies in Experiments 1 and 2.

Word Dimension								
	Critical Items		MI Items		MC Items		Neutral Items	
	RED	BLUE	BLACK	YELLOW	PURPLE	WHITE	ORANGE	GREEN
RED	96	96						
BLUE	96	96						
BLACK			32	96				
YELLOW			96	32				
PURPLE					96	32		
WHITE					32	96		
ORANGE							64	64
GREEN							64	64

*Critical items: 50% congruent, used to examine the CSE; MI items: mostly incongruent; MC items: mostly congruent, Neutral items: 50% congruent.*

#### Procedure

**FIGURE 1:**
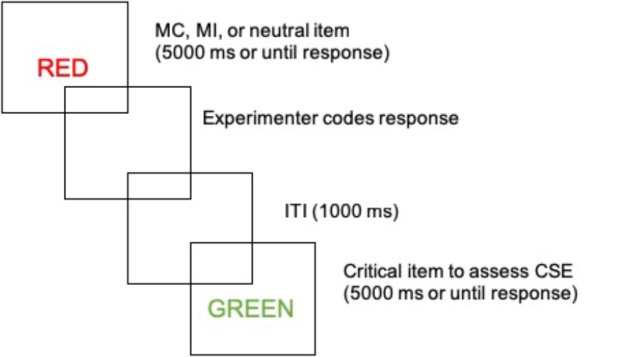
Illustration of task structure.

The experiment began with a demonstration block in which each of the eight colors were shown as colored squares and the participant was asked to name them aloud. This was followed by a 23 item practice block which mimicked the structure of the test (i.e., mostly congruent items were more frequently presented in their matching color and so forth). During practice, corrective feedback was given as necessary (e.g., “speak more loudly,” “remember to name the color not the word,” etc.). After the practice block, the test itself began, illustrated in Figure 1. The test phase consisted of 1152 trials with 12 rest breaks programmed throughout. In both the practice and test blocks, the Stroop stimulus was displayed in the center of the screen for 5000 ms or until a verbal response by the participant triggered the microphone. The participant’s response initiated a blank screen while the experimenter coded the response as correct, incorrect or microphone error (e.g., stutters, speaking too softly etc.). Once the response was coded, a 1000 ms blank screen inter-trial interval was initiated prior to the presentation of the next stimulus. The Washington University Institutional Review Board approved all procedures.

#### Analysis

To avoid the influence of outlier RTs, individual’s data were trimmed using the following method. First, microphone errors were removed followed by any valid response trial that was faster than 200 ms (presumed to be fast guesses or an undetected microphone error). Next, RTs that were faster or slower than three standard deviations from the participant’s mean were removed. Finally, we also eliminated the first trial after each break, trials that occurred after an error and any trial immediately following when the experimenter took longer than 5 s to code the response. This trimming strategy eliminated 7.4% of the total responses.

The data were then split into critical items (used for the CSE analysis) and “biased” items (MC, MI, or neutral) for an analysis of the ISPC. RTs were *z*-scored to each individual’s mean and standard deviation within each set of items to control for individual differences in overall speed and ability (Faust et al., 1999). Raw mean RTs are provided in the Supplementary Materials. Mean *z*-scored RTs were calculated for each of the critical cells for analysis. The condition means were analyzed using a Bayesian linear mixed effects model using the rjags package (Plummer, 2016). For the ISPC analysis, the condition means included congruency (congruent vs. incongruent items) and item type (MC vs. MI vs. Neutral). For the CSE analysis, the condition means reflected the three-way crossing of congruency (congruent vs. incongruent items), previous trial congruency (congruent vs. incongruent) and the previous item type (MC, MI, or neutral). In order to generate representative and stable estimates, we ran three chains of 100,000 samples from the posterior distribution and excluded the first 1,000 as burn-in for each analysis. After checking that the chains converged using the Gelman and Rubin ^ R statistic (Gelman and Rubin, 1992), we collapsed across the chains to analyze the posteriors. Mean *z*-scored RTs were analyzed as a combination of the conditions (defined above) and a random effect of subject. Each beta weight was given a broad (uninformative), normally distributed prior. Results are presented as a point estimate together with the 95% highest density interval (HDI), e.g., effect = X, HDI = Y:Z). An effect can be called “significant” if the HDI does not include zero. Finally, we provide Bayes Factor of the critical effects using the Savage-Dickey density ratio (Wagenmakers et al., 2010) as a quantification of the evidence for a given hypothesis. Conventionally, a Bayes Factor between 3.2 and 10 represents a “substantial” amount of evidence (Kass and Raftery, 1995).

### Results

#### *z*-Scored RTs

##### ISPC analysis

**FIGURE 2:**
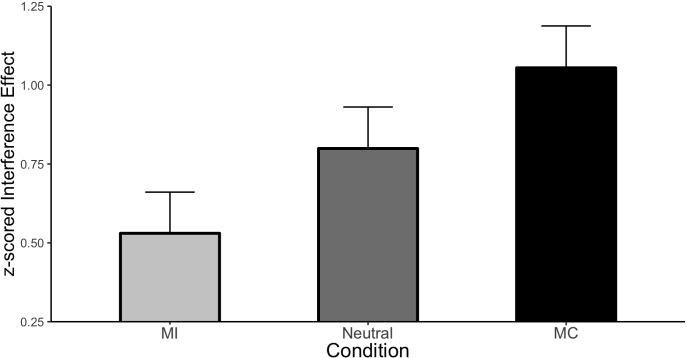
ISPC effect in Experiment 1. Error bars represent the 95% HDI.

The first and necessary step in our analysis is to demonstrate that an ISPC effect was obtained in our modified design. Condition means are displayed in Figure 2. The main effect of Stroop congruency was large and significant (Mean effect = 0.794, HDI = 0.718:0.871) indicating responses were 0.794 standard deviations slower to incongruent relative to congruent stimuli. More importantly, this effect interacted with the type of item (i.e., there was an ISPC). Specifically, relative to the neutral condition, interference was greater for the MC items (Mean effect = 0.256, HDI = 0.069:0.441) and was smaller for the MI items (Mean effect = −0.269, HDI = −0.454:−0.082). Thus, the ISPC effect is readily apparent even under these highly controlled conditions.

##### CSE analysis

**FIGURE 3:**
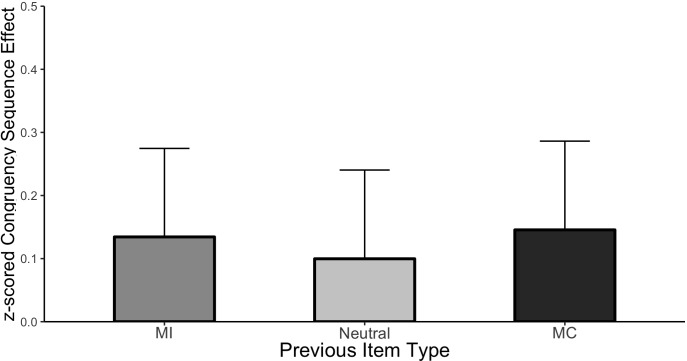
CSE in Experiment 1. Error bars represent the 95% HDI.

Figure 3 plots the CSE (post-incongruent interference minus post-congruent interference) as a function of each item type and the cell means are shown in Table 2. It is important to remember that “item type” refers to the prior trial in this analysis as the current trial was always unbiased. As before, the Stroop effect averaged across all conditions was significant (Mean effect = 0.693, HDI = 0.652:0.734). Importantly, the magnitude of interference varied as a function of prior trial congruency producing the CSE, (Mean effect = −0.126, HDI = −0.208:−0.044). However, there was no evidence of an interaction between the CSE and the prior item type indicating that the CSE was of comparable magnitude regardless of whether it followed an MC, MI, or neutral item. Specifically, the HDI of the beta weight comparing the CSE following MC items to the CSE following neutral items was wide and encompassed zero (Mean effect = −0.046, HDI = −0.248:0.151, Bayes Factor = 8.88) as did the comparison between MI and neutral (Mean effect = −0.034, HDI = −0.236:0.165, Bayes Factor = 9.21). These results indicate that although there is a clear effect of the congruency of the previous trial (the significant CSE) and the probability of the previous item being mostly congruent, incongruent or neutral (ISPC effect), these two effects did not interact.

**Table 2:** Mean *z*-scored RTs (and HDIs) for each condition in the CSE analysis of Experiment 1.

	Previous Congruent	Previous Incongruent
	**MI Items**	
Congruent	−0.441 (−0.513:−0.370)	−0.265 (−0.336:−0.194)
Incongruent	0.324 (0.253:0.395)	0.366 (0.295:0.438)

	**Neutral Items**	
Congruent	−0.400 (−0.471:−0.329)	−0.278 (−0.350:−0.208)
Incongruent	0.326 (0.255:0.397)	0.348 (0.278:0.420)

	**MC Items**	
Congruent	−0.418 (−0.489: −0.348)	−0.224 (−0.297: −0.155)
Incongruent	0.360 (0.289:0.431)	0.408 (.338 :.480)

#### Accuracy

##### ISPC analysis

For the ISPC items, the Stroop effect was significant (Mean effect = −0.036, HDI = −0.044:−0.027) indicating more errors to incongruent items relative to congruent items. Furthermore, interference was larger for MC items relative to neutral (Mean effect = −0.024, HDI = −0.044:−0.002) and also relative to MI items (Mean effect = −0.036, HDI = −0.057:−0.015). However, the MI and Neutral items did not differ from one another (Mean effect = 0.013, HDI = −0.008:0.034).

##### CSE Analysis

Looking at the critical items to assess the CSE, the Stroop effect was significant (Mean effect = −0.032, HDI = −0.039:−0.025) as was the CSE (Mean effect = 0.023, HDI = 0.010:0.036). However, none of the interactions with prior item type were significant. Specifically, the HDI of the beta weight comparing the CSE following MC items relative to neutral items was large and encompassed zero (Mean effect = 0.01, HDI = −0.019:0.046, Bayes Factor = 43.49) as was the CSE following MI items relative to neutral (Mean effect = 0.01, HDI = −0.028:0.038, Bayes Factor = 56.76).

### Interim Discussion

The primary result from this experiment is that the CSE and ISPC both produce highly reliable effects but are additive with one another. This provides initial evidence that the ISPC and CSE reflect separate and independent mechanisms. The evidence for the independence of these two factors was quite large (∼9 times in favor of the null when testing the three-way interaction, as reflected by the Bayes Factor). However, there are a number of additional reasons that might account for the null interaction we obtained. We report two additional experiments that address these possibilities. First, it is possible that we did not have a sufficiently strong CSE to detect the hypothesized interaction. Although highly reliable, the CSE is relatively small, at least when compared to the size of the overall Stroop effect. Thus, in order to both replicate our original finding and address the effect size issue, we conducted the same experiment again with an older adult sample. Older adults typically produce a larger CSE in the Stroop task relative to younger adults (Aschenbrenner and Balota, 2017) and therefore, if the null is simply due to the relatively small magnitude of the CSE, we may be more likely to detect the interaction in this population.

## Experiment 2

### Participants

A group of 32 healthy older adults (59% female; mean age = 72.7, *SD* = 4.3) were recruited from the St. Louis community. Participants were given $25 for their time and effort.

### Stimuli and Procedure

The stimuli, procedure and analysis were identical to Experiment 1. Our trimming method eliminated 6.7% of the total RTs.

### Results

#### *z*-Scored RTs

##### ISPC analysis

**FIGURE 4:**
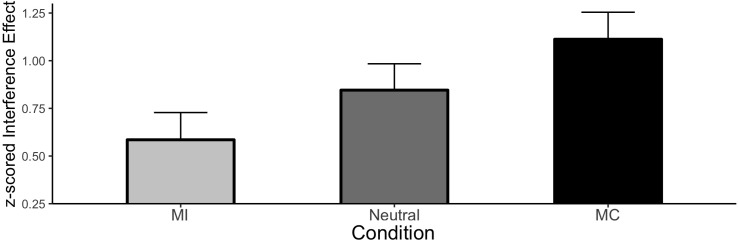
ISPC effect in Experiment 2. Error bars represent the 95% HDI.

Condition means for the ISPC effect are shown in Figure 4. As expected, there was a significant Stroop effect (Mean effect = 0.847, HDI = 0.766:0.930) indicating responses were slower to incongruent relative to congruent items. Furthermore, the interference effect was larger for MC items relative to neutral (Mean effect = 0.267, HDI = 0.067:0.465) and smaller for MI items relative to neutral (Mean effect = −0.260, HDI = −0.457:−0.06), reflecting the ISPC effect.

##### CSE analysis

**FIGURE 5:**
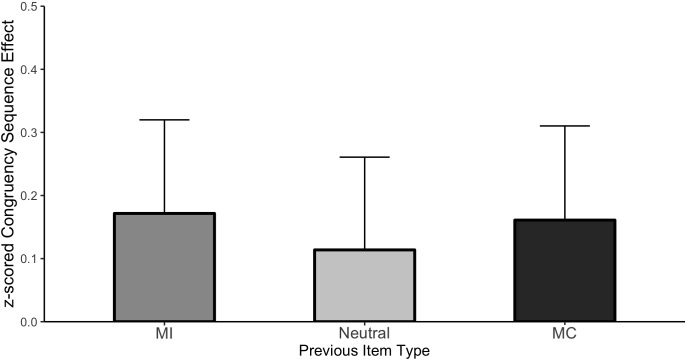
CSE in Experiment 2. Error bars represent the 95% HDI.

The CSE as a function of prior item type is shown in Figure 5 and the individual cell means are shown in Table 3. Once again, we observed a significant interference effect (Mean effect = 0.817, HDI = 0.774:0.860) as well as a significant CSE (Mean effect = −0.149, HDI = −0.234:−0.062) indicating smaller interference effects following an incongruent stimulus. Critically, the CSE did not interact with the prior item type. Specifically, the HDI for the difference between prior MI and prior neutral trials was wide and included zero (Mean effect = −0.058, HDI = −0.268:0.149, Bayes Factor = 8.09) as was the HDI of the difference between prior MC and prior neutral items (Mean effect = −0.047, HDI = −0.256:0.159, Bayes Factor = 8.51).

**Table 3:** Mean *z*-scored RTs (and HDIs) for each condition in the CSE analysis of Experiment 2.

	Previous Congruent	Previous Incongruent
	**MI Items**	
Congruent	−0.495 (−0.570:−0.421)	−0.336 (−0.411:−0.262)
Incongruent	0.431 (0.356:0.505)	0.418 (0.343:0.493)

	**Neutral Items**	
Congruent	−0.493 (−0.568:−0.419)	−0.322 (−0.396:−0.247)
Incongruent	0.370 (0.296:0.444)	0.427 (0.353:0.501)

	**MC Items**	
Congruent	−0.402 (−0.476:−0.328)	−0.355 (−0.430:−0.281)
Incongruent	0.481 (0.408:0.557)	0.368 (0.293:0.442)

#### Accuracy

##### ISPC analysis

In the analysis of accuracy rates, the Stroop effect was significant (Mean effect = −0.022, HDI = −0.028:−0.015). Interference was larger for the MC items relative to neutral (Mean effect = −0.020, HDI = −0.035:−0.004) but the MI and neutral items did not differ from one another (Mean effect = 0.005, HDI = −0.010:0.021).

##### CSE analysis

In the analysis of the CSE items, the Stroop effect was reliable (Mean effect = −0.017, HDI = −0.021:−0.013) but there was no CSE (Mean effect = 0.002, HDI = −0.01:0.006). Furthermore, the CSE following MC items did not differ from neutral items (Mean effect = −0.002, HDI = −0.02:0.018, Bayes Factor = 101.88) nor did the MI items differ from neutral (Mean effect = −0.004, HDI = −0.023:0.015, Bayes Factor = 93.03).

### Discussion

We replicated our initial findings of additive effects of the CSE and ISPC in an older adult cohort. The ISPC itself was large and significant which suggests that control settings are being modulated on those trials. Furthermore, the CSE itself was also significant indicating responses are being adjusted based on the *congruency* of the prior trial regardless of whether it was an MI, MC or neutral item. Importantly, a simple ANOVA confirmed that the cross-experiment Age by CSE interaction was reliable, F(3,186) = 3.13, *p* = 0.03, indicating that older adults produced larger CSEs compared to younger adults, collapsed across ISPC conditions, replicating the recent Age × CSE interaction that was reported by Aschenbrenner and Balota (2017). Moreover, the present replication and extension of Experiment 1 to an older adult sample again suggests that the CSE and ISPC reflect distinct mechanisms.

Before reaching such a conclusion, there is one final possibility regarding these additive effects that remains to be evaluated. Specifically, although we motivated the current experiments under the notion that the ISPC reflects an adjustment in control processes (i.e., when an MI item is encountered control is rapidly increased), an important alternative account of the ISPC is one of associative stimulus-response learning. For example, if BLUE is most frequently presented in the color red (hence is a mostly incongruent item), participants can learn that when the stimulus is the word BLUE they should respond with “red” (Schmidt and Besner, 2008). Indeed, a number of studies have suggested that once this contingency bias is experimentally controlled for, ISPC effects disappear (Schmidt and Besner, 2008; Schmidt, 2013; Hazeltine and Mordkoff, 2014). Thus, under this scenario the ISPC may not be an issue of control but rather a reflection of associative learning and therefore one many not expect to observe an interaction between the ISPC and CSE.

Of course, it is important to note that we included “neutral” items in our ISPC design, that is, items that were always 50% congruent. Therefore, if the MI or MC items invoked an associative learning mechanism, one would still have expected to obtain an interaction whereby the neutral items (which must be resolved via attentional control) interact with the CSE but not the biased items (which may reflect associative learning). This presents some initial evidence that associative learning processes may not be the entire story in the first two experiments. However, to further address this important concern, we conducted a final experiment in which we attempted to minimize the contribution of an associative learning mechanism. We do this by drawing on the Associations as Antagonists to Top-Down Control (AATC) hypothesis proposed by Bugg (2014). Specifically, Bugg argued that contingency biases typically produce the ISPC under most circumstances but when contingencies are accounted for, conflict adaptation processes then take over. For example, in an experiment when associative learning processes would be expected to be quite strong (e.g., when MI items only occur in one other color, red always in BLUE), no evidence of conflict adaptation was observed (there was no list-wide proportion congruency effect). However, when associative learning was lessened by simply increasing the number of response options, (e.g., when the word blue could occur in RED or GREEN), conflict adaptation was again observed. Thus, when reliable S-R associations can form (see blue respond RED), modulations of control are minimal whereas when the associations are not reliable (see blue respond either RED or GREEN) control adjustments are more likely prevail. Thus, as a final attempt to address the concern that associative learning processes are producing the ISPC in our studies, we followed Bugg (2014) by increasing the stimulus-response set such that each word is paired with *two* possible colors rather than just *one*.

## Experiment 3

### Participants

Sixty-six participants were recruited from the Psychology Department undergraduate research pool (67% female; mean age = 19.5, *SD* = 1.2). Our power analysis showed that this sample size gave 95% power for a meaningful (greater than three) Bayes factor in favor of the alternative hypothesis (assuming a moderate effect size) and 82% power to obtain a meaningful Bayes factor in favor of the null (assuming effect size of 0).

### Stimuli

The stimuli were the same as those used in Experiments 1 and 2. However, as shown in Table 4, the frequency of presentation of each item has changed. Specifically, we eliminated the neutral items and now presented 3 MC items and 3 MI items which were counterbalanced and rotated across participants. In this way, we reduced the ability to rely on associative learning to resolve the interference on the biased items.

**Table 4:** Stimuli frequencies in Experiment 3.

	Critical Items		MI Items			MC Items		
	RED	BLUE	BLACK	YELLOW	PURPLE	WHITE	ORANGE	GREEN
RED	36	36						
BLUE	36	36						
BLACK			16	24	24			
YELLOW			24	16	24			
PURPLE			24	24	16			
WHITE						48	8	8
ORANGE						8	48	8
GREEN						8	8	48

*Critical items: 50% congruent, used to examine the CSE; MI items: mostly incongruent; MC items: mostly congruent.*

### Procedure

The procedure was very similar to Experiments 1 and 2 with the exception of the stimulus configurations detailed above and that only 528 trials were presented with 36 practice items and 7 pre-programmed breaks. These changes were implemented to reduce the length of the experiment. We increased our sample size to compensate for these lower trial counts and also to increase our overall power. The analysis and trimming procedures were otherwise identical to the previous two experiments and 9.2% of RTs were identified as outliers and removed prior to analysis.

### Results

#### *z*-Scored RTs

##### ISPC analysis

**FIGURE 6:**
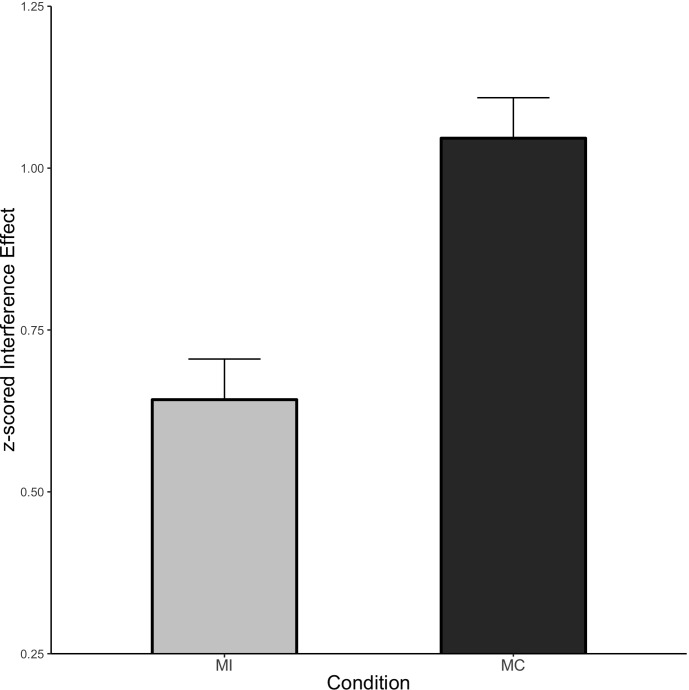
ISPC effect in Experiment 3. Error bars represent the 95% HDI.

The condition means for the ISPC analysis are shown in Figure 6. There was a large and significant Stroop interference effect (Mean effect = 0.844, HDI = 0.800:0.888) which interacted with item type. Specifically, interference was larger for MC items relative to MI items (Mean effect = 0.404, HDI = 0.316:0.492). Thus, even though the associative learning confound was minimized in this design, we are still able to detect a large ISPC effect.

##### CSE analysis

**FIGURE 7:**
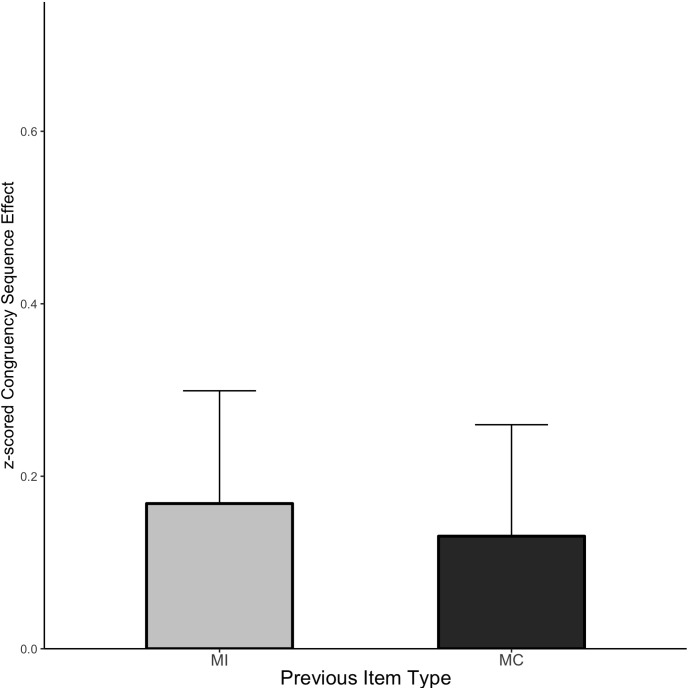
CSE in Experiment 3. Error bars represent the 95% HDI.

The CSE means are displayed in Figure 7 and the cell means are shown in Table 5. The interference effect was reliable (Mean effect = 0.872, HDI = 0.825:0.919) and interacted with prior trial congruency (Mean effect = 0.149, HDI = 0.056:0.242) reflecting the standard CSE. However, there was still no evidence of an interaction with the prior item type (Mean effect = −0.038, HDI = −0.221:0.150, Bayes Factor = 9.73).

**Table 5:** Mean *z*-scored RTs (and HDIs) in each condition for the CSE analysis of Experiment 3.

	Previous Congruent	Previous Incongruent
	**MI Items**	
Congruent	−0.543 (−0.609:−0.476)	−0.375 (−0.441:−0.309)
Incongruent	0.468 (0.402:0.534)	0.467 (0.402:0.533)

	**MC Items**	
Congruent	−0.489 (−0.555:−0.423)	−0.335 (−0.401:−0.269)
Incongruent	0.392 (0.327:0.459)	0.416 (0.351:0.483)

#### Accuracy

##### ISPC analysis

For the ISPC items, the average Stroop effect was significant (Mean effect = −0.038, HDI = −0.048:−0.028) and this effect interacted with the prior item type (Mean effect = 0.031, HDI = 0.011:0.053) such that interference was larger for MC items relative to MI items, producing the ISPC.

##### CSE analysis

For the CSE items, the average Stroop effect was significant (Mean effect = −0.034, HDI = −0.044:−0.025) but the effect was not modulated by prior trial congruency (congruent vs. incongruent, Mean effect = −0.009, HDI = −0.028:0.011). Furthermore, the CSE did not interact with the prior item type (MC items vs. MI items, Mean effect = 0.013, HDI = −0.026:0.051, Bayes Factor = 41.25).

### Discussion

The results of Experiment 3 once again clearly demonstrated the presence of both a robust ISPC effect and a CSE but no hint of an interaction between these two factors. This replicates our prior experiments under conditions that minimize associative learning as a possible mechanism for the ISPC. Thus, the control settings engaged on Trial N-1 to produce the ISPC do not appear to differentially influence the interference effect on the subsequent trial (the CSE).

## General Discussion

The primary aim of this work was to examine the relationship between two purported markers of dynamic adjustments in attentional control, the ISPC and the CSE. The main finding, replicated across three experiments, was that although there was both a robust ISPC and a CSE, these two manipulations did not interact. In other words, the CSE examined on Trial N was of a comparable magnitude regardless of the congruency bias of the stimulus on Trial N-1. Indeed, the Bayes Factor was quite large (∼9) in support of this null interaction, within each experiment. Additive factors logic therefore suggests that the mechanisms responsible for producing the change in interference reflected in the ISPC are not the same as the mechanisms producing the CSE, at least in the present experiments.

These results are consistent with a recent study that indirectly tested a similar idea. Specifically, Crump et al. (2018) used an attention capture paradigm that included an ISPC manipulation. In supplementary analyses, it was shown that sequential effects (i.e., the CSE) did not interact with the ISPC. We critically build on this work by a) including a set of well-controlled, contingency minimized “critical” items on which to assess the CSE in order to avoid the various confounds that hinder analysis of the CSE (e.g., Duthoo et al., 2014a) and b using a standard, vocal-response Stroop task, the quintessential measure of attentional control, in which most studies have explored both CSE and ISPC effects.

As already mentioned, both the ISPC and the CSE have been thought to reflect rapid and dynamic adjustments in attentional control processes. To the extent that these manipulations influence the same mechanism, one would expect a design that manipulates both would produce an interaction. Specifically, consider a congruent, MI item. Typically, the MI manipulation would produce an increase in control, due to the frequency manipulation (i.e., the ISPC) but the item would also be expected to reduce control due to the fact that it is congruent (producing the CSE). A priori, one would expect the CSE to be canceled out or at least minimized in this scenario, producing a statistical interaction. The robust additive pattern between the ISPC and CSE obtained in the current series of experiments would appear to call into question any mechanistic explanation of the CSE that relies on singular dynamic adjustments in control processes. Indeed, these results seem to suggest that the CSE is not a control modulation phenomenon at all, but rather may result from a more general mechanism that induces trial by trial changes in the recruitment of the specific operations that are employed to achieve a given task based on recent experience. In other words, the specific operations that are engaged on Trial N (whatever they may be) are informed by which operations were employed on the prior trial.

This idea is embodied in the pathway priming account of Stroop performance noted earlier (Aschenbrenner and Balota, 2015). That is, the use of a particular pathway, either color or word processing, is primed for use depending on the extent to which that pathway could be used on the prior trial. When a congruent trial was just processed, the word reading pathway is relied upon to a greater extent on the following trial, since it was a useful pathway to facilitate processing. Of course, in the context of conflict tasks such as the Stroop task, “reliance” on a given pathway is also a reflection of control processes. That is, attentional control dictates the degree of activation that propagates along any given pathway. While we are suggesting that pathway priming is independent from control processes *per se*, consistent with the additive effects obtained in the present study, we acknowledge that the overlap in mechanisms makes totally disentangling these processes rather difficult. Therefore, the extent to which local cross-trial changes in the Stroop task match those from other domains (e.g., visual word recognition or short-term memory scanning) provides a useful avenue to understand general mechanisms of dynamic (due to previous trials) adjustment of stimulus response configurations to accomplish task goals.

As noted earlier, it is interesting to note that our interpretation of the CSE is consistent with an established literature on cross-trial effects in other cognitive domains. For example, it has been repeatedly shown that in the lexical decision task, the speed to identify a stimulus as a word or nonword depends on the perceptual and response characteristics of both the current and previous trial (Balota et al., 2013; Masson and Kliegl, 2013). Specifically, if two adjacent trials are perceptually degraded, RTs are faster compared to when the perceptual clarity changes across trials. Moreover, if the lexical status of the previous trial is the same as the current trial (e.g., two “nonword” targets in a row), there are large effects of response congruency. We have proposed that this finding reflects the system adjusting to prepare to process the same, salient characteristics across trials (Balota et al., 2018). Importantly, however, large manipulations of variables known to influence lexical processing (e.g., word frequency) on Trial N are not influenced by previous trial characteristics (degradation or lexicality) which is similar to the current experiments where the CSE on trial N is not influenced by the ISPC on Trial N-1. Similar findings have been recently demonstrated in a diverse array of tasks including noun/verb judgments and short term memory scanning (Aschenbrenner et al., 2017) and speeded word naming (Zevin and Balota, 2000; Reynolds and Besner, 2005) suggesting that cross-trial influences is a rather general mechanism and not tied to tasks that presumably tap attentional control, such as the Stroop task.

The present study has many strengths including the replication of a theoretically important null effect across multiple experiments and samples, however a few limitations are worth mentioning. First, we focused only the influence of the immediately preceding trial. While it is fair to say this is the standard approach in the field, this approach does minimize the cumulative influence of multiple serial trials and may not accurately reflect the time course of control. For example, Jiménez and Méndez (2013) examined the CSE as a function of runs of 1, 2, or 3 sequential trials of the same congruency and they showed the congruency effect increased as the number of presented congruent trials increase but the effect decreased when numerous incongruent trials were presented. However, because the CSE is greatest from trial N-1 to trial N, the current study afforded the strongest test of a single trial dynamic adjustment in control. Second, we began these investigations under the assumption that the ISPC effect is due to modulations in attentional control that occur post-stimulus onset (Jacoby et al., 2003). However, such an interpretation is still under fierce debate in the literature (Bugg and Crump, 2012; Schmidt, 2018). As the contingencies of the items in our experiments still varied across the ISPC manipulations (even in Experiment 3) whether our results successfully precluded the contributions of S-R learning processes cannot be fully determined. At a minimum, however, these results can serve as a starting point for additional experimentation that can more cleanly separate these component processes.

## Conclusion

In summary, the ISPC and CSE were robustly additive across three distinct experiments. This pattern suggests that the CSE reflects an independent, response adjustment system and may not be related to adjustments in attentional control *per se*, at least as reflected by the ISPC effect. Hence, these results provide evidence of multiple distinct forms of response dynamics in the premier measure of attentional control, the Stroop task. The similarity of cross-trial effects in other standard cognitive tasks that do not demand high levels of control further question the standard interpretation of the CSE primarily reflecting dynamic changes of attentional control.

## Ethics Statement

This study was carried out in accordance with the recommendations of Institutional Review Board at Washington University in St. Louis with informed consent from all subjects. All subjects gave verbal informed consent in accordance with the Declaration of Helsinki. The protocol was approved by the Washington University in St. Louis Institutional Review Board.

## Author’s Note

The ability to rapidly direct attention to important aspects of the environment while ignoring distracting information is a critical cognitive skill. This paper investigates the relationship between different variables that are thought to engage these attentional processes. The results show there are multiple, independent factors that can aid or hinder the ability to focus attention on important, but possibly less salient, information in the face of distractors.

## Author Contributions

AA collected and analyzed the data, and drafted the manuscript. DB edited the manuscript for intellectual content.

## Conflict of Interest Statement

The authors declare that the research was conducted in the absence of any commercial or financial relationships that could be construed as a potential conflict of interest.
