# Supplementary material for: Additive Effects of Item-Specific and Congruency Sequence Effects in the Vocal Stroop Task
Source: Front Psychol. 2019 Apr 24;10:860. doi: 10.3389/fpsyg.2019.00860 (PMC6491926; doi:10.3389/fpsyg.2019.00860)
Supplement: Supplementary file 1 [file Table_1.DOCX]

Supplementary Material

Supplementary Table 1: Raw RT means (and SDs) of the CSE analysis in Experiment 1.

|  | **MI Items** | |
| --- | --- | --- |
|  | Previous Congruent | Previous Incongruent |
|  |  |  |
| Congruent | 595 (90) | 615 (84) |
| Incongruent | 692 (107) | 697 (112) |
|  | **Neutral Items** | |
|  | Previous Congruent | Previous Incongruent |
|  |  |  |
| Congruent | 598 (86) | 613 (90) |
| Incongruent | 690 (110) | 694 (107) |
|  | **MC Items** | |
|  | Previous Congruent | Previous Incongruent |
|  |  |  |
| Congruent | 596 (88) | 620 (88) |
| Incongruent | 695 (110) | 702 (105) |

Supplementary Table 2: Raw RT means (and SDs) of the CSE analysis in Experiment 2.

|  | **MI Items** | |
| --- | --- | --- |
|  | Previous Congruent | Previous Incongruent |
|  |  |  |
| Congruent | 699 (106) | 724 (106) |
| Incongruent | 847 (155) | 844 (137 |
|  | **Neutral Items** | |
|  | Previous Congruent | Previous Incongruent |
|  |  |  |
| Congruent | 697 (90) | 728 (105) |
| Incongruent | 838 (142) | 845 (140) |
|  | **MC Items** | |
|  | Previous Congruent | Previous Incongruent |
|  |  |  |
| Congruent | 715 (112) | 718 (106) |
| Incongruent | 855 (138) | 837 (133) |

Supplementary Table 3: Raw RT means (and SDs) of the CSE analysis in Experiment 3.

|  | **MI Items** | |
| --- | --- | --- |
|  | Previous Congruent | Previous Incongruent |
|  |  |  |
| Congruent | 590 (73) | 610 (76) |
| Incongruent | 708 (104) | 710 (109) |
|  | **MC Items** | |
|  | Previous Congruent | Previous Incongruent |
|  |  |  |
| Congruent | 597 (75) | 614 (81) |
| Incongruent | 701 (111) | 706 (110) |
